# Anti-K1 (Kell) Antibody Expressed in Maternal Breastmilk: A Case Report of a Neonate with Multiple Intrauterine Transfusions and Postnatal Exposure to Kell Antibody in Maternal Breastmilk

**DOI:** 10.1155/2017/6927813

**Published:** 2017-03-05

**Authors:** Patrick DeMoss, Mohamed Asfour, Kelly Hersey

**Affiliations:** ^1^Blair E. Batson Children's Hospital, Department of Pediatrics, Jackson, MS, USA; ^2^Department of Pathology, University of Mississippi Medical Center, Jackson, MS, USA; ^3^Blair E. Batson Children's Hospital, Department of Neonatology, Jackson, MS, USA

## Abstract

Hemolytic disease of the fetus and newborn is a common consideration in newborn medicine, especially among the jaundiced. Maternal breastmilk provides numerous benefits to the infant, including nutrition and immunologic factors. Here, we present an infant who received three intrauterine transfusions for anemia secondary to anti-K1 (Kell), anti-C, and anti-e antibodies and whose maternal breastmilk tested positive for anti-Kell antibodies. The infant required another transfusion at 4 weeks of life for anemia. We review the pathophysiology of anti-Kell antibodies, the immunology of breast milk, and the intersection of these two topics.

## 1. Introduction

Neonatology is more unique among the fields of medicine in that it often illustrates the intersection among several domains of clinical and laboratory medicine, as this case demonstrates. Hemolytic disease of the fetus and newborn (HDFN) is a common consideration in medicine of the newborn, especially among the jaundiced. There are many antibodies responsible for HDFN, most famously anti-RhD. Soon after birth, the mother begins expressing colostrum and breastmilk. Breastmilk has numerous benefits and is an essential source of nutrition and immunologic protection for the newborn. Here, we present the intersection of hemolytic disease of the fetus and newborn with breastmilk by the discovery of anti-Kell antibody in maternal breastmilk supply.

## 2. Case Description

Boy K's mother was referred to Maternal Fetal Medicine for previous dichorionic diamniotic twin gestation with demise of one twin at 8 weeks gestation and maternal anti-e, anti-K1 (Kell), and anti-C antibodies discovered during the prenatal antibody screen. Father tested positive for the Kell antigen. Middle cerebral artery flow velocity was monitored with weekly ultrasounds. Maternal anti-K1 titers were positive at 22 weeks gestation (titer of 2048 with a score of 99), 28 weeks gestation (titer of 1024 with a score of 103), and 31 weeks gestation (titer of 1024 with score of 103). At 25 weeks gestation, the middle cerebral artery peak systolic velocity was 72.08 cm/second and periumbilical transfusion was performed with type O, Rh positive, K1-, C-, and e-antigen negative, leukoreduced, CMV-safe, sickle-cell negative, irradiated washed packed red blood cells. Ultrasound monitoring was increased to biweekly measurements of middle cerebral artery flow velocity. A second and third transfusion were required at 28 and 31 weeks gestation. By 34 weeks gestation, the middle cerebral artery flow velocity remained elevated at 63.8 cm/second. After a course of betamethasone (corticosteroids) the infant was delivered by scheduled Caesarean-section at 35 weeks gestation. There was no evidence of fetal hydrops on any of the prenatal ultrasounds.

Infant required no resuscitation at birth. Apgars were 8 and 9 at 1 and 5 minutes, respectively. Birth weight was 2.3 kilograms. His initial hematocrit was 44% with a 1.9% reticulocyte count. Mother's blood type was O+, as was the infant's. Direct antiglobulin testing at birth on the baby was positive for anti-Kell and anti-C antibodies. His total bilirubin was 7.46 mg/dL by 12 hours of age with no direct bilirubin, and phototherapy was started. Follow-up total bilirubin at 24 hours of age was 7.24 mg/dL and 5.95 mg/dL by 36 hours of age. Phototherapy was stopped after 36 hours and the follow-up total bilirubin remained acceptable for age at 6.95 mg/dL. Total bilirubin peaked at 13.19 mg/dL on the fourth day of life.

Enteral feedings began on the second day of life when it was determined that an exchange transfusion would not be necessary. Initial feedings with 22 calorie per ounce premature infant formula continued until mother was able to pump and begin breast feedings shortly thereafter. After consent was obtained, maternal milk was tested and confirmed positive for anti-Kell antibodies but was not tested for other antibodies.

At the age of 4 weeks, he was seen by hematology for an abnormal newborn screen showing hemoglobin FS. At that visit, his physical exam was notable for significant conjunctival pallor but no jaundice noted to his mucous membranes, sclera, or skin. On laboratory studies, he was anemic (hemoglobin 6.0 g/dL and 10.8% reticulocyte count). His antibody screen was again positive with his plasma showing anti-C antibodies and the red blood cell eluate showing anti-C and anti-Kell antibodies. He was transfused 50cc of packed red blood cells. Up to that point, mother had been breastfeeding him about 3 ounces every 3 hours. Follow-up labs at 4, 8, and 16 weeks after transfusion showed hemoglobin 8.7 g/dL with 3.2% reticulocyte count, hemoglobin 10.2 g/dL with 3.1% reticulocyte count, and hemoglobin 9.7 g/dL with 3.5% reticulocyte count, respectively. Between 8 and 16 weeks after transfusion, mother switched him to formula feedings.

## 3. Discussion

Hemolytic disease of the fetus and newborn (originally termed hemolytic disease of the newborn) was first clinically described by Levine et al. in 1941 concerning the Rh group [1]. Other antigens have since been associated with hemolytic disease. The Kell red cell antigen includes 24 different members, with at least eight antigens associated with hemolytic disease of the fetus and newborn (HDFN), with the K1 antigen frequently associated with severe disease [2, 3]. The Kell antigen is expressed in the bone marrow and fetal liver and expressed only on progenitor and mature red blood cells [4–6]. The K1 antigen, in particular, is found in 9% of the Caucasian population and 2% of the African-American population [7].

Anti-Kell antibodies become a particular issue if the mother is sensitized and the baby is expressing the antigen. Incidence of the maternal anti-Kell antibody is reported at 1/1,000 pregnancies [8, 9]. In thirteen pregnancies with a Kell-sensitized mother and a Kell-positive infant, five (38%) had poor outcomes, and, in eighteen pregnancies with affected Kell-sensitized mothers and Kell-positive infants, ten (55%) displayed moderate to severe disease [8, 10]. Mother may be sensitized to the Kell antigen by two predominant mechanisms: transfusion from a Kell-positive donor or by fetal hemorrhage of Kell-positive blood in to the maternal circulation. Transplacental sensitization occurred in almost half (51%) of a series of 65 pregnancies, with 45% having a history of maternal transfusion [10].

The nature of the anemia when Kell antigens are present is different from the more classic scenario with anti-RhD isoimmunization [11, 12]. Anti-RhD isoimmunization is characterized by extravascular hemolysis, after maternal IgG antibodies tag foreign fetal antigens, making them prime targets for splenic macrophages that hemolyze the red blood cells extravascularly [3]. In anti-RhD isoimmunization, there is the typical inverse relationship between reticulocyte count and hemoglobin, which is not characteristic of anti-Kell isoimmunization [12].

The anemia from anti-Kell isoimmunization seems to be a combination of destruction of early red blood cells and suppression of red blood cell production. Anti-Kell antibodies specifically inhibit red blood cell progenitors, as the antigen is characteristically expressed on immature red blood cells [13]. This is supported clinically by a suppression of red blood cell activity. In anti-Kell isoimmunization, there is reduced reticulocytosis and erythroblastosis with lower amniotic fluid bilirubin compared to the anti-D group [11, 12]. Doppler ultrasonography is used to noninvasively monitor fetal anemia [14], with subsequent intrauterine blood transfusions as rescue therapy as needed.

Breastmilk has numerous benefits for the newborn by providing optimal protein and fat for weight gain, neural factors for brain development, factors for intestinal growth and repair, and antibodies for immune development [15]. The predominant antibody in colostrum (>90%) and later breast milk is secretory IgA, followed by IgM and IgG [16–18]. Different classes of antibodies serve disparate roles in the immune system and also elicit variable responses within the body. IgG antibody is vitally important in utero for developing a robust immune system, as this antibody readily crosses the placenta and bolsters immunity for the first several months of a newborn's life [19]. Secretory IgA is an antibody produced by the mother in response to antigens presented to her in the gut lumen and then ultimately passed along to the newborn through breastmilk via the enteromammaric link [20].

The anti-Kell antibody is predominantly IgG and consequently should be present in colostrum and early breast milk. While the relative proportions of immunoglobulins remain fairly constant, the absolute load of IgG likely falls to a nadir between 1 and 2 weeks postpartum, with levels steady thereafter. The value of this decline seems to be variable, but significant declines of 90% have been demonstrated in studies [17, 21]. However, another study did not show IgG levels dropping postpartum but instead remaining constant [16]. Maternal colostrum in this case tested positive for anti-Kell antibody. Unfortunately, serial antibody titers of breastmilk were not measured nor was a paired maternal serum sample sent. With testing in progress, a literature review showed that anti-Kell antibodies were discovered in murine breastmilk as well [22].

“Physiologic” anemia of newborn is typically seen at 6 weeks of age in premature infants (7–10 g/dL) [23]. The direct coombs test is used to answer the following question: is IgG or complement bound to the patient's red cell membrane? The indirect coombs test is used to detect antibodies in the patient's sera by using red blood cells with known antigens on their surfaces [24]. Several questions arise from his laboratory findings: can we attribute his anemia to anti-C hemolysis? Is suppression of erythroblastosis from anti-Kell? Are anti-C and anti-Kell together? Or is a constellation of physiologic anemia coupled with anti-C and anti-Kell antibodies? Is the positive direct coombs result a function of continued expression via maternal breast milk or is the test measuring red blood cells that were tagged in utero?

Given the aforementioned evidence that anti-Kell induces a profound reticulocytopenia, its role in this clinical scenario is difficult to elucidate since his reticulocyte count was 10.8%. Anti-C antibody is of the Rhesus type and as such would induce extravascular hemolysis [3]. Visual assessment of jaundice can be highly accurate in ruling out significant hyperbilirubinemia but not for assessing serum levels if hyperbilirubinemia is present [25]. Unfortunately, serum bilirubin was not determined at the hematology visit, but his physical exam was reassuring enough to not order this measure.

In summary, anti-Kell antibodies were present in mother's breastmilk, further confirming the observation of Santhanakrishnan et al. [22]. Future directions could address the serial titers of these antibodies in breastmilk coupled with maternal serum titers. The infant's hemoglobin and bilirubin could also be measured concurrently to assess if there is any anemia or hemolysis. This patient further reinforces that breastmilk is an immunologically rich source of nutrition that serves as a transition from intrauterine to extrauterine life.

## Abbreviations

HDFN: Hemolytic disease of fetus and newborn.
